# Global Learning for Health Equity: Assessing Pilot Grants as a Tool to Advance the Strategy

**DOI:** 10.5334/aogh.5155

**Published:** 2026-04-17

**Authors:** Virginia Rowthorn, Shadae Chambers, Yolanda Ogbolu

**Affiliations:** 1 University of Maryland School of Graduate Studies, 621 West Lombard Street, Baltimore, MD 21201, USA; 2 University of Maryland School of Nursing Baltimore, Maryland, USA

**Keywords:** global learning, health equity, reciprocal innovation, community‑based organizations, global–local partnerships, implementation science

## Abstract

*Background:* Persistent health and healthcare inequities in the United States, rooted in systemic racism and power imbalances, continue to drive poor health outcomes for marginalized communities. Global learning, also termed reciprocal innovation, has emerged as a promising strategy for addressing these inequities by adapting ideas across national contexts through mutually beneficial partnerships.

*Objective(s):* To describe the Global Learning for Health Equity (GL4HE) Pilot Grant Initiative as a strategy to advance health equity in US communities, examining the characteristics of participating organizations, the utility of the GL4HE Framework, and the role of funding, mentorship, and peer learning.

*Methods:* This evaluation drew on multiple forms of descriptive information generated during Network implementation, including an external evaluation report, grantee final narrative reports, mentor–mentee feedback surveys, and observations documented during technical assistance sessions and convenings. These materials were qualitatively analyzed to identify recurring observations related to strengths, barriers, organizational readiness, and the perceived usefulness of Network supports, allowing for an integrated understanding across sources.

*Findings:* Through the GL4HE Pilot Grant Initiative, seven organizations implemented global learning projects addressing varied health equity challenges. Grantees valued the GL4HE Framework for providing a shared language and conceptual roadmap but noted gaps in actionable guidance. Seed funding, intensive mentorship, and convenings were critical to progress. Deep community engagement and reciprocal cross-cultural exchange also emerged as key enablers of success. Major challenges included administrative burdens, partner identification, travel logistics, and concerns about long-term scalability and sustainability.

*Conclusions:* Pilot grants, when paired with mentorship, peer learning, and a guiding framework, can effectively catalyze global learning for health equity. To maximize impact, future efforts should provide more operational tools, streamline administrative processes, and invest in sustained, community-centered partnerships. Global learning represents a viable and important approach for advancing equitable, innovative solutions to complex health challenges in the United States.

## Introduction

Persistent health inequities in the United States, driven by entrenched power imbalances, continue to shape disproportionately poor health outcomes for marginalized communities [1]. These disparities contribute to higher rates of premature death and disability. As one response, there is growing interest in learning from other countries’ successful approaches to improving health equity. This strategy, called *global learning* for purposes of this article, is defined as “the practice of engaging with, exchanging, and adapting health equity‑promoting ideas and interventions between communities in ways that foster implementation benefits that are reciprocal and beneficial to both” [2]. Also called “reciprocal innovation” [3], global learning is a bidirectional, community‑centered approach that emphasizes knowledge exchange across national borders and avoids extractive or imposed practices [4, 5].

By the early 2000s, reverse innovation (as it was known at the time) was increasingly promoted as a strategy to strengthen health systems in high‑income countries and as a practical mechanism for introducing solutions to places, such as the United States, facing steep healthcare costs, systemic inefficiencies, and persistent inequities [6, 7]. Many of these calls for global learning can be traced to Global North researchers who engaged with health care systems in low‑ and middle‑income countries as part of the global effort to address the HIV/AIDS crisis [5]. Openness to learning from other nations has steadily expanded as the hold of colonial assumptions and US exceptionalism has weakened, creating room to recognize that other countries often demonstrate equal or greater innovation, particularly in achieving more equitable and universal health care [8, 9].

When used for health equity purposes (For the purposes of this article, we use the World Health Organization’s definition of health equity, which holds that health equity is achieved “when everyone can attain their full potential for health and well‑being.”) [10], global learning can be a transformative approach for communities in the United States by helping them look beyond traditional strategies to novel solutions created by peer communities overseas. Working with international communities fosters reciprocal relationships that, in addition to providing innovative solutions, can foster solidarity across borders and support communities in leading their own development and shaping their futures.

**Figure 1:**
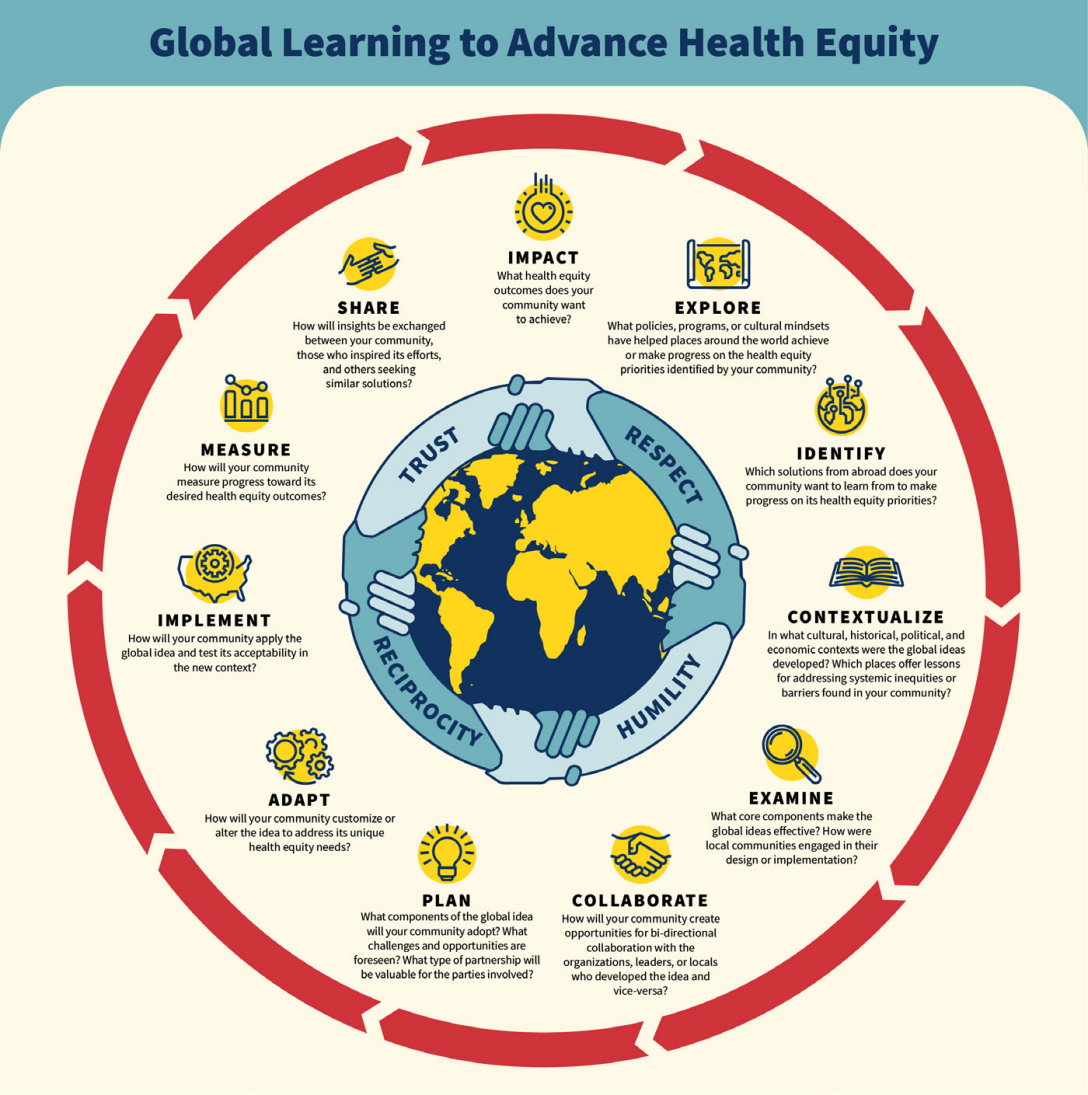
The GL4HE Framework.

The Global Learning for Health Equity Network (GL4HE Network), launched in 2020, is a national platform that connects practitioners, researchers, and communities who are applying global ideas to advance health equity in the United States. The Network is led by a consortium of six academics, public health officials, and health care providers with experience in global learning, affiliated with the University of Maryland, Baltimore (UMB), Brigham and Women’s Hospital and Navajo Nation, Montefiore Health System and Albert Einstein College of Medicine, The Corner Health Center, and the Athens City‑County Health Department. The GL4HE Network has defined global learning, developed a Framework to guide global learning (see Figure 1), and conducted a Pilot Grant Initiative to support global learning in seven communities in the US. Grantees of the Pilot Grant Initiative utilized the Framework as a foundational guide in implementing their initiatives.

This manuscript presents a comprehensive review of the Pilot Grant Initiative, examining the characteristics of participating community‑based organizations (CBOs) that supported effective engagement with global learning. It also evaluates the contributions of the GL4HE Network—including the Framework, seed funding, and expert mentoring—to grantee success, highlighting supports that were especially valuable and those requiring further refinement. Collectively, the Pilot Grant Initiative generated practical lessons about how global learning can be strengthened and scaled in US settings, clarifying what is needed to advance its use in efforts to address health equity challenges.

## Methodology

This assessment used a qualitative approach grounded in the Consolidated Framework for Implementation Research (CFIR) [11]. It drew on multiple forms of descriptive information generated throughout the implementation of the GL4HE Network. Sources included grantee final narrative reports, mentor–mentee feedback surveys, written summaries of observations from technical assistance (TA) sessions and Network convenings, and the external evaluation report commissioned for the initiative that synthesized findings from focus groups.

These materials were systematically reviewed with deductive thematic analysis to infer implicit and explicit meanings and identify consistent themes in global learning experiences. Statements across the different information sources that reflected key CFIR constructs, such as partnerships and connections, innovation adaptability, and available resources [11], were highlighted and assigned the code that represented the CFIR construct they most closely aligned with. These reflections were then extracted and categorized to identify salient patterns across sources, such as commonly reported strengths, barriers to implementation, indicators of organizational readiness, and perceptions of Network supports. Findings were developed by comparing recurring observations within each category and by noting areas of convergence and divergence. This approach allowed the authors to integrate perspectives from grantees, evaluators, and Network leaders to provide a comprehensive descriptive account of Network implementation.

## The GL4HE Framework

The GL4HE Network Framework (Figure 1) was developed as an early product of the GL4HE Network and describes stages through which communities implement global learning projects and the questions communities should ask themselves in each of the stages [12]. Prior work by the authors identified 24 documents in the public domain that provide guidance, checklists, or frameworks to support or overcome barriers to global learning. Four of the included frameworks are specifically designed to promote health equity [13]. However, none reflected the fluid and varied reality of global learning in practice, in which communities enter the process at different, non‑linear stages and require tailored support to progress along those stages. To address this gap, the GL4HE Network created a framework with 11 stages grouped in five buckets:

1. Exploration—identifying the health equity challenges of the community and what impact is desired, scanning and identifying global ideas, and building community awareness of possibilities.
2. Pre‑implementation (sometimes called adaptation/planning)—assessing transferability of the identified intervention, adapting design, and building partnerships and community readiness.
3. Implementation—rolling out the adapted intervention in the local context.
4. Evaluation—assessing the intervention’s effectiveness, process, fidelity, and equity outcomes.
5. Dissemination/Sustainment—sharing lessons, scaling, sustaining, and engaging in bidirectional or multi‑directional learning with originating community and others.

Although the framework reflects the life cycle of global learning and a structured roadmap, the authors’ prior research and experience demonstrated that communities launch their global learning journeys at different stages along the framework [12]. For instance, a community may have already identified one or more health equity concerns in their population and only come to global learning when other “local” options have failed or when someone proposes an intervention that has been successful overseas for a similar challenge. Another community may be open to global learning and yet not sure how to start the process of identifying an international intervention or how to be in communication with the community where the intervention was developed. The purpose of the GL4HE Framework is to help those interested or involved in global learning to visualize the process and potential next steps.

## GL4HE Pilot Grant Initiative

The Pilot Grant Initiative was launched by the GL4HE Network in 2023 to provide funding and support to organizations interested in global learning for health equity. A wide range of organizations were encouraged to apply—such as non‑governmental organizations, CBOs, public health departments, and university centers—as long as they had a demonstrated history of community engagement. Applicants had to identify a health equity challenge in their US community, their organization’s current stage of global learning, plans for community engagement, and—if applicable—a global initiative or project for adaptation. Each application was independently reviewed by three GL4HE experts who evaluated the applicant’s goals for global learning, history of community engagement, feasibility, and alignment of budget with project goals. The composite ratings of these criteria were averaged across the three reviewers to create an overall score that was used to rank and ultimately determine the top seven proposals to be selected for pilot grant funding.

Grants of $50,000 were made to four CBOs, two university centers, and a public health department (see list in Table 1). Three of the applicants were ready to collaborate with their identified global partner, and one was already planning to adapt a specific global initiative. The remaining organizations were early in the global learning process, either at the Impact, Explore, or Identify phase, and ready to take the next steps with funding and mentorship. The 2‑year grant funding was accompanied by mentorship from the GL4HE Network leadership team, virtual TA sessions, and three convenings for community building and training. At the end of the grant term, grantees provided final reports.

**Table 1:** Overview of Pilot Grant Projects.

PROJECT TITLE	LOCAL ORGANIZATION(S)	INTERNATIONAL PARTNER(S)	HEALTH EQUITY ISSUE	KEY ACHIEVEMENTS
**4 Pillars: Centering Women’s Access through Agency**	The Corner Health Center & CORDUSA* (Washtenaw County, Michigan)	CORD (Himachal Pradesh, India)	Barriers to healthcare, education, and employment for women aged 12+	Fully implemented model with member‑led program development and execution
**Global Exploration of Age‑Friendly and Aging Supportive Communities**	Athens City‑County Health Department (Ohio)	Anton Trstenjak Institute of Gerontology and Intergenerational Relations (Slovenia)	Place‑based and aging‑related barriers to health and well‑being	Acceptance into WHO Global Network for Age‑Friendly Cities and Communities; identified potential ideas for implementation in Ohio
**Global Learning to Improve Palliative Care in Tanzania and USA**	University of Maryland School of Nursing (Baltimore, MD)	Evangelical Lutheran Church of Tanzania	Limited access to quality palliative care services in both urban United States and rural Tanzanian settings	Co‑created potential solutions in both countries
**The Hood Exchange Inaugural Racial Healing Cohort**	The Hood Exchange(Atlanta, GA)	POS (Perfector of Sentiments) Foundation (Accra, Ghana)	Black mental health, belonging, and wellness among formerly incarcerated young Black adults	Reconnection to African diasporic roots; increased exposure to and understanding of different forms of healing
**Global Learning for Adolescent Sexual and Reproductive Health Equity**	Montana State University (Bozeman, Montana)	University of Nairobi; Jomo Kenyatta University; St. Paul’s University (Kenya)	Sexual and reproductive health inequities among rural and indigenous adolescents in Montana	Bidirectional exchange of sexual and reproductive health challenges and current practices
**Global Learning in Addressing Trauma—Cambodia/US Immigrant Communities**	The Cambodian Family (Santa Ana, California)	Transcultural Psychosocial Organization Cambodia; Eye Movement Desensitization and Reprocessing—Cambodia; Caritas; APCT Life Science Center; Royal University of Phnom Penh	Trauma and mental health stigma among Cambodian communities in the United States	Integrated traditional practices into US services; piloted evidence‑based training in Cambodia
**BMORE Global @ Community Walk‑Through Theater**	Community Walk‑Through Theater (Midtown Edmondson Community—West Baltimore, MD)	ACT (Act, Change, Transform) Nairobi (Kenya)	Social isolation, mental health, chronic illness, food access, housing, intergenerational disconnect	Assessed community interest in global learning; identified global partner to inspire creative health communication strategies

*Chinmaya Organization for Rehabilitation and Development by Undertaking Sustainable Activities.

## Cross‑Cutting Lessons Learned

The Pilot Grant Initiative yielded a number of valuable conclusions that deepen our understanding of how global learning can be effectively implemented to promote health equity in the United States. The conclusions clarify the use of the GL4HE Framework as well as identify practical strategies, common challenges, and enabling conditions that can support health equity promoters in applying global learning more effectively. By distilling lessons from the experiences of participating communities, this work lays the groundwork for more accessible, scalable, and impactful use of global learning as a tool for advancing equity in diverse local contexts.

### A. Value of GL4HE framework to grantees

The GL4HE Framework was designed as a structured roadmap for global learning. Previous scholarship identified 24 guidance documents, checklists, and frameworks to support or overcome barriers to global learning, four of which are specifically designed to promote health equity [13]. Lacking from these frameworks, however, is an acknowledgment of the multiple steps involved in global learning and the questions that should be considered along the way. The GL4HE Network developed its Framework based on prior research demonstrating that organizations engage in global learning in a variety of different ways and starting at different entry points.

Grantees noted the value of the framework and commended it because it provides a clear structure to identify and adapt new global ideas. However, one key finding that emerged from the Pilot Grant Initiative was that the framework primarily offered guidance at a conceptual or high‑level stage. While it was effective in introducing the underlying concepts to community members and other stakeholders, some noted that it lacks specific, actionable direction to assist grantees in progressing from one phase to the next. For example, for grantees in the “Explore” stage, the framework did not provide concrete mechanisms for identifying potential initiatives abroad or establishing connections with originating communities. This particular transition, from Explore to Identify, proved especially challenging for several grantees and highlighted not only limitations of the framework itself but also broader difficulties inherent in the practice of global learning.

To address these challenges, the Network plans several supportive strategies. Although numerous databases of promising global innovations exist [14, 15, 16], many lack up‑to‑date contact information and details and some grantees expressed discomfort at the thought of “cold” contacting organizations without an introduction or other intermediary step. Therefore, the Network will develop a curated database of vetted international initiatives and partners to help organizations identify credible opportunities more efficiently. In addition, creating “matchmaking” or mentorship programs that connect US‑based CBOs with experienced global organizations would better facilitate relationship‑building and knowledge exchange. The Network will also offer workshops focused on cross‑cultural engagement, partnership development, and global learning practices, equipping CBOs with the skills needed to navigate international collaboration. Finally, establishing regional hubs or peer learning groups would enable organizations to share experiences, strategies, and resources, strengthening their collective ability to move from exploration to identification.

### B. Mentorship and technical assistance

Grantees were given one‑on‑one mentorship for the duration of the grant through regular meetings to provide advice as needed on all elements of their projects. Mentees reported that this personalized support was an invaluable element to project success, keeping them on track and helping to bridge unforeseen gaps.

*“Our project coach helped us test our thinking about various aspects of the project’s implementation and provided a key support in connecting us to various resources.” (Pilot Grant Initiative grantee)*

Grantees who had established relationships with a global partner needed less mentorship than those in earlier stages. For organizations with existing global partners, mentorship primarily involved mentees seeking strategic and logistical guidance from mentors who had previously navigated the global learning process. In contrast, for those in the “Explore” phase, mentorship evolved into a more collaborative arrangement—resembling a partnership or co‑directorship—focused on critical organizational decisions related to international innovation, community engagement, and partnership development. As noted earlier, this distinction underscores that supporting CBOs in progressing from exploration to active implementation necessitates intensive and structured forms of support.

Another supportive component of the Pilot Grant Initiative was TA. At the outset of the Pilot Grant Initiative, Network leaders organized three initial TA sessions, with additional sessions scheduled in response to feedback obtained during mentorship meetings:

1. Storytelling Through Images
2. Sharing Stories Using Collective Wisdom to Make Improvement
3. Implementation Science
4. History and Culture of Navajo Nation
5. Community Partnership
6. Sustainability
7. Updates on the Progress
8. Sustainability and Seeking New Funding Opportunities

Despite the alignment of these online TA sessions with topics identified by grantees as areas of need, attendance was relatively low. While this may partly reflect the demanding schedules of participating grantees, mentors also observed that broad, generalized guidance was considerably less useful than individualized, one‑on‑one support, particularly given the complexity and contextual specificity of global learning initiatives. Nonetheless, the grantees deemed both TA and mentorship as critical elements to enhancing their understanding of the application of global learning and implementation science to their work and broadening their existing perceptions of health equity and community engagement, as evidenced by the following quote:

*“Guidance on sustainability planning, measurement strategies, and cross‑site partnership development strengthened our capacity to think beyond immediate activities toward long‑term system change.” (Pilot Grant Initiative grantee)*

### C. Convenings and peer learning

Three convenings were held over the course of the Pilot Grant Initiative: an initial meeting at the UMB; a midterm convening hosted by one of the grantees in the Navajo Nation, New Mexico; and a final session at UMB, which also served as a public forum on global learning. During these gatherings, grantees presented their projects and progress, participated in TA activities through breakout sessions, and, at the concluding convening, engaged in panel discussions on global learning scholarship and implementation featuring organizations beyond the GL4HE Network. International partners were not funded to attend these convenings; however, the pilot grants did support reciprocal site visits, enabling US teams to travel to their international partner communities and, in turn, allowing international partners to visit the United States as part of the collaborative learning process.

A remarkable feature of these convenings was their ability to foster new collaborations and connections among health equity promoters who might not otherwise have crossed paths.

*“Observing different approaches, perspectives, and cultural practices encouraged us to critically examine our assumptions about what works in our own community and adapt innovative strategies from other countries. This exposure broadened our understanding of community engagement, strengthened our methodology, and reinforced the importance of contextualizing global interventions for local impact.” (Pilot Grant Initiative grantee)*

Feedback from participants indicated that the opportunity to join a network of peers fostered an environment of mutual learning and problem‑solving, allowing participants to exchange practical insights, reflect on challenges, and develop a sense of collective purpose. This provided both emotional encouragement and professional reinforcement, both resources that were particularly vital for small CBOs undertaking the complex and often isolating work of developing global learning initiatives, as it *“reminds us that we’re not alone, and … reinforces our commitment to shared goals.” (Pilot Grant Initiative grantee)*

### D. Community engagement facilitates success

#### Community engagement in US community

Essential to the success of global learning is the existence, or deliberate cultivation, of trust within the community at all stages. Community members were engaged in the process through listening sessions and focus groups to identify health equity challenges in the community, determine support for global learning, and brainstorm potential solutions to address local needs. Projects were most impactful when communities were not just participants but co‑creators actively participating in every step of the process, including traveling internationally to study the global idea firsthand. This inclusive, relational approach fostered a sense of ownership, ensured cultural relevance, and empowered communities as agents of change. One example is the Hood Exchange cohort, comprised of formally incarcerated men, who actively explored and engaged in culturally immersive activities and wellness workshops during a visit to their partner organization in Ghana. The 4 Pillars Model also fostered community agency by enabling participants to autonomously create agendas and lead their own programming.

Among the CBOs, those that were more established with a longer history of engagement in the community, as well as those with more staff and funding, were better able to mobilize community support for every stage of global learning. The Cambodian Family, for instance, has been working with immigrant families in Orange County, California for four decades, serving more than 8,600 clients annually. The deep trust that they had already established with Cambodian immigrant families in the United States catalyzed their ability to forge strong partnerships in Cambodia and create a seamless bridge between domestic and international interventions.

#### Cross‑cultural exchange with international community

Another core component of global learning is cross‑cultural exchange, typically facilitated through partnerships with international organizations that share aligned goals. Many grantees engaged in reciprocal site visits, traveling abroad and hosting their partners in the United States, to foster mutual understanding and culturally immersive learning experiences. These exchanges enabled participants to reflect on the similarities and differences between global and local challenges, identify effective practices, and adapt culturally grounded approaches to their own contexts. Grantees consistently identified these exchanges as transformative, not only for project development but also for cultivating respect, brainstorming, and shared insight.

These exchanges highlighted an important but rarely discussed aspect of global learning: the process does not typically begin with identifying an international innovation for US adaptation and then pursuing cross‑cultural exchange. Rather, it is often the cross‑cultural exchange based on personal or organizational connections that then leads to the sharing of successful strategies that can be adapted in the United States. To illustrate, it was not until their site visit to Slovenia that the Athens City‑County Health Department identified various Slovenian community models that could be valuable for the aging population in Ohio.

The goal of global learning is to leverage cultural exchange and bidirectional learning with international communities not only to inform adaptations in US communities, but also to contribute back to originator communities as those adaptations evolve. In practice, innovations will circulate in multiple directions over time, with some strategies adapted for US contexts and others returning to international partners in modified or enhanced forms in an ongoing, additive, and mutually beneficial process. However, this cannot and should not be achieved without building connections and trust among stakeholders in order to avoid an extractive or exploitive approach to knowledge generated by others.

The framework is explicit in outlining the need to engage in global learning in the context of mutually beneficial partnerships. This priority was achieved by the grantees in several ways, including funding the travel of international partners to the US community, engaging in regular videoconferences to share ideas, preparing jointly authored manuscripts, and planning joint projects (outside of the funded project) in the future. For example, Montana State University is collaborating with their partners in Kenya on a publication that explores factors influencing adolescent sexual and reproductive health in rural Kenya. Likewise, the Cambodian Family is planning to develop cross‑national training pathways to ensure sustained trauma‑informed and culturally grounded care in both the United States and Cambodia.

## Challenges and Barriers to Pilot Grant Success

The grantees encountered multiple challenges during the implementation of their projects. Programmatic challenges included identifying appropriate international partners, connecting with new international partners, coordinating collaboration across organizations, and aligning project objectives with broader institutional missions of involved organizations. Logistical difficulties related to travel and visas for incoming international visitors to the United States were the most frequently cited barriers. One recurring issue involved the complexities of collaborating with academic institutions, particularly the protracted and cumbersome contracting and invoicing procedures, which often resulted in delayed payments and hindered project momentum. Streamlining administrative processes or channeling funds through less restrictive entities may alleviate these burdens and enhance efficiency especially for grassroots organizations. The GL4HE Network views these challenges as valuable opportunities for growth in the continued evolution of this work.

While some grantees praised the Pilot Grant Initiative for providing a conceptual framework and shared language for implementing global learning, others expressed a need for more concrete guidance on operationalizing the framework in practice. Future efforts will therefore focus on strengthening both the conceptual understanding and the practical application of global learning, ensuring that community leaders possess the necessary readiness and capacity for effective implementation.

Equally important is the dissemination of Pilot Grant Initiative outcomes to audiences most likely to benefit from them, for example, other CBOs. To this end, the network will explore inclusive and accessible dissemination strategies, including resource documentation and storytelling methods tailored to diverse audiences. Finally, the most pressing concern among grantees was the sustainability of their initiatives. In the context of global learning, sustainability operates on two interconnected levels: the long‑term continuity of the work within the US‑based community and the durability of the binational relationships that make this work possible. These forms of sustainability are distinct but mutually reinforcing, and both are essential to the authors’ conception of global learning. At the same time, both forms of sustainability are challenging to achieve and merit further study to understand how best to cultivate and support them over time. Addressing this concern will require ongoing education on the value of global learning as a powerful tool in the health equity toolkit, a tool worthy of resources and support.

## Implications and Recommendations

The Pilot Grant Initiative offers several insights for funders, implementers, and policymakers seeking to advance equitable and sustainable global learning projects. First, the pilot grants underscore the importance of contextualized support for CBOs. Small‑ and mid‑sized CBOs often lack international networks and administrative infrastructure, which makes global collaboration logistically and financially demanding. Funders and policymakers should therefore recognize that equitable participation in global learning requires not only grant funding but also capacity‑building investments, such as mentorship, administrative assistance, and accessible technical guidance tailored to community contexts.

For implementers, the pilot grants illustrate that successful global learning depends on sustained relationship‑building, flexibility, and shared learning between US‑based and international partners. Programs that integrate structured mentorship, peer exchange, and locally driven project design are better equipped to navigate cultural and institutional complexities.

In terms of scaling and sustaining these efforts, several recommendations emerge. Funders should consider multi‑year or phased funding models that allow for iterative learning and deeper partnerships over time. Establishing a network of practice, a sustained community of grantees, mentors, and partners with a topical or geographic focus, could institutionalize peer support and shared learning across projects. Streamlining contracting and payment processes, or routing funds through community‑trusted intermediaries, would further reduce administrative burdens on smaller organizations.

To support long‑term sustainability, it will be important to embed global learning into existing local priorities and funding streams, such as public health, education, and community development initiatives. This approach allows US governmental organizations, CBOs, and NGOs to look beyond the United States for practical solutions to domestic challenges, particularly those affecting marginalized communities. As part of this work, relationships with international partners will naturally exist along a continuum; some collaborations may be short‑term exchanges, while others may deepen over time. The goal is mutual learning and locally relevant problem‑solving, not a prescriptive or burdensome partnership model. Policymakers can reinforce this integration by creating incentives for cross‑sector collaboration and by recognizing global learning as a core component of civic and workforce development.

## Conclusion

The Pilot Grant Initiative demonstrated that when community‑based organizations are given the resources, relationships, and confidence to engage with partners beyond their immediate contexts, they generate innovative solutions grounded in local realities. While these projects were designed to address local challenges, their broader value lies in the insights they offer to other communities confronting similar problems. When shared and thoughtfully adapted, locally developed strategies can inform responses in other settings and contribute to a wider process of collective problem‑solving.

The experiences of these pilot projects highlight both the promise and the practical challenges of translating global learning into sustained action. They also underscore a critical lesson: no single country or community holds a monopoly on effective solutions. Communities around the world are continuously developing approaches to health, equity, and sustainability. Engaging with these experiences expands the range of ideas available to address complex challenges and strengthens the capacity of community‑based organizations to respond creatively and effectively.
